# Sex and age differences in mice models of effort-based decision-making and anergia in depression: the role of dopamine, and cerebral-dopamine-neurotrophic-factor

**DOI:** 10.1007/s00213-023-06430-7

**Published:** 2023-08-18

**Authors:** Paula Matas-Navarro, Carla Carratalá-Ros, Régulo Olivares-García, Andrea Martínez-Verdú, John D. Salamone, Mercè Correa

**Affiliations:** 1 Àrea de Psicobiologia, Campus de Riu Sec, Universitat Jaume I, Castelló 12071 Castelló de la Plana, Spain; 2 Behavioral Neuroscience Div., Psychological Sciences, University of Connecticut, Storrs, CT 06269-1020 USA

**Keywords:** Sex, Age, Effort, Vigor, Dopamine, Neurotrophic factors, Operant, Running wheel, Mice

## Abstract

Mesolimbic dopamine (DA) regulates vigor in motivated behavior. While previous results have mainly been performed in male rodents, the present studies compared CD1 male and female mice in effort-based decision-making tests of motivation. These tests offered choices between several reinforcers that require different levels of effort (progressive ratio/choice task and 3-choice-T-maze task). Sweet reinforcers were used in both tasks. In the operant tasks, females worked harder as the task required more effort to access a 10% sucrose solution. Although males and females did not differ in preference for 10% vs 3% solutions under free concurrent presentation, females consumed more of the 10% solution when tested alone. The operant task requires a long period of training and changes in the DA system due to age can be mediating long-term changes in effort. Thus, age and sex factors were evaluated in the T-maze task, which requires only a short training period. Both sexes and ages were equally active when habituated to the running wheel (RW), but females consumed more sweet pellets than males, especially at an older age. Both sexes had a strong preference for the RW compared to more sedentary reinforcers in the 3-choice-T-maze test, but older animals spent less time running and ate more than the young ones. The DA-depleting agent tetrabenazine reduced time running in older mice but not in adolescents. Cerebral-dopamine-neurotrophic-factor was reduced in older mice of both sexes compared to adolescent mice. These results emphasize the importance of taking into account differences in sex and age when evaluating willingness to exert effort for specific reinforcers.

## Introduction

One characteristic of motivated behavior is that a high degree of work is often expended in order to achieve a goal. Thus, high levels of activity, effort, vigor, and persistence are essential features of motivated behavior (Salamone and Correa 2002, 2012). Strong changes in these activational aspects of motivation can be seen in many psychopathologies. For example, reduced exertion of effort for goal directed activity is often seen in people with major depression (Caligiuri and Ellwanger 2000; Salamone and Correa 2012, 2022; Tylee et al. 1999; Demyttenaere et al. 2005). In fact, some of the most common symptoms of depression are energy-related dysfunctions such as slowness, tiredness, listlessness, and apathy (Tylee et al. 1999; Salamone and Correa 2012). Many people with major depression have fundamental deficits in exertion of effort, and effort-related decision-making (Treadway et al. 2012b), which is marked by a reduced likelihood of selecting high effort alternatives (Treadway et al. 2012a; Yang et al. 2014). Moreover, fatigue seems to be a symptom that is marked by clear sex differences among depressed patients. It has been observed that “somatic” symptoms such as fatigue are more common among women (Bjornelv et al. 2011; Dekker et al. 2007). In addition, women diagnosed with depression usually express greater lack of activity than men (Poutanen et al. 2009; Breslin et al. 2006). Furthermore, the incidence of depression increases in older adulthood, and somatic complaints are one of the aspects that present a higher occurrence (Fonda and Herzog 2001; Balsis and Cully 2008; Sutin et al. 2013). In fact, symptoms like lack of energy are more common in advanced age (Liao and Ferrell 2000). The term fatigue or energy-related dysfunction in this context does not refer to muscle or peripheral metabolic fatigue, but rather, to “central fatigue,” which is a subjective lack of physical and/or mental energy that is thought to be related to brain mechanisms (Schwid and Murray 2005). Fatigue and self-reported lack of energy can collectively be described as anergia. Anergia biases individuals towards sedentary lifestyles and reduced exertion of effort (Salamone et al. 2016a; Kuppuswamy 2017).

Tests of effort-based decision-making in rodents are useful as models of motivational symptoms of depression (Salamone et al. 2003, 2007, 2015, 2016a, 2016b; Salamone and Correa 2012, 2022). Motivational fatigue, anergia, and effort-based decision-making can be studied using tasks that offer choices between alternatives that vary in terms of their effort requirements. Thus, animals have to choose between high-effort options leading to highly valued reinforcers vs. low-effort options that procure a less-valued reward. Interference with nucleus accumbens (Nacb) dopamine (DA) modifies the selection of high-cost high-reward alternatives and biases animals towards low-effort choices, but nevertheless leave fundamental aspects of hedonic reactivity, food consumption, and preference intact (Salamone and Correa 2002, 2012, 2022; Salamone et al. 2016b; Salamone et al. 2018; Salamone et al. 2022). The progressive ratio (PROG)/chow feeding choice task is a commonly used procedure in which rats are given the option of engaging in PROG lever pressing reinforced by preferred pellets vs. approaching and consuming a less preferred laboratory chow that is concurrently available in the chamber (Randall et al. 2012, 2014; see reviews Salamone and Correa 2002, 2012, 2022). This operant procedure has a version using sucrose reinforcement for rats, which does not require food restriction (Pardo et al. 2015, 2020; SanMiguel et al. 2018). Using this highly effort-demanding task, it has been demonstrated that drugs that reduce or interfere with DA function increase selection of the less demanding option, increasing also consumption of the less effort requiring reinforcer, and drugs that increase DA function produce the opposite effect (see reviews in Salamone et al. 2007, 2016a, 2016b, 2018, 2022).

Several of these effort-based tests have been adapted or developed specifically for mice (Pardo et al. 2012; Correa et al. 2016, 2020), which is important for obtaining cross-species validation and studying the broader variety of genetic manipulations done in this species. Mice are a very active species that show a high level of preference for vigorous physical activities, such as wheel running (Sherwin 1998). Thus, in a T-maze-choice task in which animals can choose between different types of reinforcers with different vigor requirements, one of the options is running in a running wheel (RW), and the other more sedentary options are palatable food intake or sucrose drinking and non-social neutral odors (Correa et al. 2016, 2020; López-Cruz et al. 2018; Carratalá-Ros et al. 2020, 2021a, 2021b; Presby et al. 2020). Using this effort-based choice test, it has been shown that DA D2 receptor antagonism (Correa et al. 2016, 2020), and DA depletion in mice (López-Cruz et al. 2018; Carratalá-Ros et al. 2020, 2021a, 2021b), reduced selection of RW activity but concurrently increased more sedentary behaviors such as sweet food eating or sucrose drinking. On the other hand, drugs that increase DA transmission in Nacb such as bupropion (Randall et al. 2014), a catecholamine transport blocker used to treat depression, produced the opposite effect (Carratalá-Ros et al. 2021b).

Although the vast majority of these studies have been performed with male animals, it is fundamental to use female as well as male animals in studies of effort-based decision-making. In the present studies, we aimed to characterize baseline performance in male and female mice in a series of paradigms that evaluate the activational component of motivation, either under no choice conditions or in paradigms that evaluate willingness to engage in effortful behaviors when different options are available. This knowledge will help in the selection of experimental conditions for each sex to better assess potential therapeutic manipulations. Thus, the outbred CD1 strain of mice was used to assess males and females on different tasks of effort-based choice: a lever pressing/sucrose drinking choice procedure, and a RW/sucrose feeding/odor choice task, as well as behavioral activation in a RW, and sucrose or high carbohydrate consumption under no choice conditions. This strain has been previously used in our studies of behavioral activation and effort-based decision-making in which we have assessed the effect of DA depletion and antagonism (Pardo et al. 2012, 2013; López-Cruz et al. 2018; Correa et al. 2016, 2018, 2020; Carratalá-Ros et al. 2020; Yang et al. 2020), and the potential of some types of antidepressants or stimulant drugs for reversing their anergic effects (Pardo et al. 2012, 2013; López-Cruz et al. 2018; Carratalá-Ros et al. 2021a, 2021b, 2023). Antidepressant drugs that have a main effect on blocking DA uptake, such as bupropion, and adenosine A2A receptor antagonists that are colocalized with DA D2 receptors and act on the same postsynaptic cascade in an opposite way to DA antagonists, reverse the anergic effects of DA depletion or DA D2 receptor antagonists (Pardo et al. 2012, 2013; López-Cruz et al. 2018; Carratalá-Ros et al. 2021b; Carratalá-Ros et al. 2023). However, antidepressants such as fluoxetine, which act mainly as SERT blockers do not improve, and even exacerbate, the anergic effects of DA depletion in male mice (Carratalá-Ros et al. 2021a; Carratalá-Ros et al. 2023). This dissociation has also been observed in rats (Randall et al. 2014; Yohn et al. 2016; Presby et al. 2021).

Another important aspect in the characterization of sex differences in effort-based decision-making is age. DA neurons are thought to be particularly vulnerable to aging processes (McWain et al. 2022). For example, DA receptor expression and DA release and activity in the Nacb reaches its highest point during mid-adolescence and young adulthood respectively, declining during adulthood (Burke and Miczek 2013; Karkhanis et al. 2019; Huang et al. 1995; Pitts et al. 2020; Santiago et al. 1993; Stamford 1989). Age also has an impact on neurotrophic factors relevant for neuronal function (Lommatzsch et al. 2005). Evidence shows that the brain-derived neurotrophic factor (BDNF) is an important biomarker for the pathogenesis of depression. In depressed people, its levels are significantly decreased (Carniel and da Rocha 2021). Moreover, women have lower BDNF levels than males, and the levels of BDNF in plasma decrease with increasing age (Lommatzsch et al. 2005). In rodent models, it also has been found that with increasing age, BDNF levels decrease in different brain regions (Katoh-Semba et al. 1998). The cerebral dopamine neurotrophic factor (CDNF), a neurotrophic factor found in DA neurons, has been demonstrated to promote the survival of DA terminals (Lindholm et al. 2007; Lindahl et al. 2020; Eremin et al. 2021), and has been related to anti-inflammatory and antiapoptotic properties (Eremin et al. 2021), processes that increase due to age (Conde and Streit 2006).

Thus, in the last series of studies, we evaluated the impact of age and DA depletion in both sexes on the selection of vigorous activities that compete with other more passive activities. We also determined if basal levels of CDNF are different between sexes and if this neurotrophic factor changes in Nacb Core across the range of ages studied in the behavioral experiments.

## Materials and methods

### Animals

A total of 186 CD1 *outbred* mice were used in these studies (*N*=92 females and *N*=94 males). For the operant procedures, animals started training at 5 weeks of age and they finished at 21 weeks of age. For the T-maze experiments, one group of animals started when they were adolescent (corresponding to 8 weeks of age), while the other group were middle-aged adults (21 weeks of age at the beginning of the experiment). For the CDNF experiments, the range of ages was a bit broader, i.e., 6, 16, and 29 weeks old at the moment of perfusion. All animals were the same sex and age housed in groups of three or four per cage, with standard laboratory rodent chow available during all the experimental procedures. In the home cage, mice had water *ad libitum*, except for the sucrose preference and operant tests in which mice were water restricted to 4.0 ml/day/mouse for females and 5.0 ml/day/mouse for males in order to avoid satiation immediately before the testing sessions. Body weight was monitored daily, and this restriction allowed animals to gain weight at the rate as non-restricted animals of the same age housed in the colony. Lights were on from 08:00 to 20:00 h. The temperature of the colony was kept at 22 ± 2°C. All procedures were covered by a protocol approved by the Institutional Animal Care and Use Committee of the Universitat Jaume I. All experimental procedures complied with directive 2010/63/EU of the European Parliament and of the Council and with the “Guidelines for the Care and Use of Mammals in Neuroscience and Behavioral Research,” National Research Council 2003, USA. All efforts were made to reduce the number of animals used and to minimize animal suffering.

### Pharmacological agents

Tetrabenazine (TBZ, Cymit Quimica SL, Spain) was dissolved in a vehicle solution of 0.9% saline (80%) plus dimethyl sulfoxide (DMSO 20%, final pH 5.5) and administered 2 h before testing. The dose of 8.0 mg/kg of TBZ was selected based on previous studies demonstrating that, in mice, this dose is the most efficient at depleting DA in ventral striatum and at inducing behavioral effects in these paradigms (López-Cruz et al. 2018; Carratalá-Ros et al. 2020, 2021a, 2021b). Vehicle and TBZ were administered intraperitoneally (IP).

### Behavioral testing procedures

**Fig. 1:**
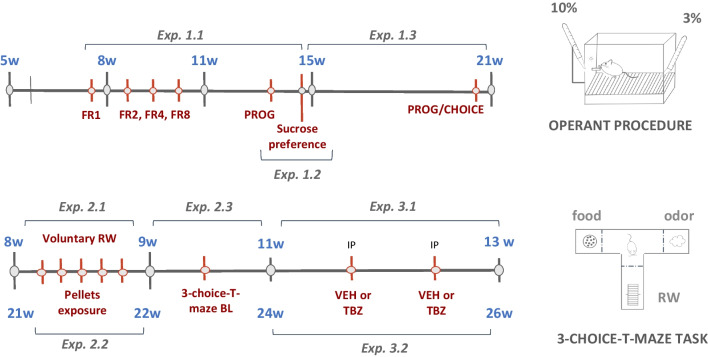
Schematics with the timeline and age of the animals in the different experiments

All behavioral procedures started 2h after the light-period onset. A soft light was used to illuminate the behavioral test room and external noise was attenuated (Fig. 1).

#### Operant Procedures

Behavioral sessions were conducted in modular operant conditioning chambers (24 × 20 × 19 cm; Med Associates). The lateral panel of each chamber was equipped with one retractable response-lever that was connected to a retractable spout of a liquid sipper bottle containing a 10.0% sucrose concentration. Completion of the ratio was indicated by a dim yellow light right above the lever immediately followed by reward availability (access to the retractable sipper). Only for the “PROG/Choice” procedure, there was a second spout of a liquid sipper bottle containing a less preferred 3.0% sucrose solution that was concurrently and continuously available on the opposite lateral panel. The start of the session was signaled by turning on the house light, and the session lasted 15 min. Operant training was performed once a day, 5 days per week. Mice were initially trained to lever press on a FR1 schedule solution for 3 weeks, and then shifted to FR2, FR4, and FR8 1 week for each schedule. After that, animals were exposed to a progressive ratio (PROG) schedule for four additional weeks. In this procedure, the ratio started at FR2 and increased exponentially every time 3 reinforcers were obtained (FR2 × 3, FR4 × 3, FR8 × 3, FR16 × 3, FR32 × 3, FR64 × 3, FR128 × 3…). The PROG schedule included a 2-min time-out, when mice did not press the lever on that time, the response lever would retract and animals could not get the reinforcer. Then, for the PROG/Choice training, the less-preferred, less-effort demanding reinforcer (3.0% sucrose) was introduced and it was concurrently available during the operant session. Mice were trained for an additional 6 weeks on that schedule. Electrical inputs/outputs of each chamber were controlled by an IBM compatible PC (Med-Associates software). Data presented correspond to the average of the last week for each schedule.

#### Sucrose preference test

After the completion of the PROG training and before the PROG/Choice training (animals were 15 weeks old), the same animals that were exposed to the operant procedure were placed individually in standard holding cages with free *ad libitum* access to two graduated cylinder tubes containing 3.0% or 10.0% sucrose drinking solutions during 30-min sessions for two consecutive days (weekend days). A longer period ensured less error in the measurement of the total fluid consumed. The left-right position of the tubes were randomly assigned to different mice in order to control possible side preferences. At the end of the session, bottles were immediately removed from the holding cage, and the remaining liquid was registered (Exp. 1.2). Sucrose concentrations were chosen based on pilot studies for sucrose preference tests from our laboratory. Data presented correspond to the average of both days.

#### Voluntary running in the RW test

Mice were introduced individually in polypropylene boxes similar to home cages with a stainless steel voluntary RW (Ugo Basile S.R.L., Germonio, VA, Italy). RWs were equipped with two magnets and they were connected to a magnetic counter that registered the number of turns. Behavioral sessions took place during 5 consecutive days, in sessions lasting 15 min each (Exp. 2.1.).

#### T-maze RW-Sweet pellets-Odor Choice task

The T-maze apparatus consists of a central area that leads to three arms (25 cm L × 11 cm W × 30 cm H). In each of them, there was a different stimulus that require different levels of activity; a cotton ball soaked with fruit odor, high carbohydrate pellets (TestDiet, 50% sucrose, 45mg each), or a RW (based on López-Cruz et al. 2018). Tests were conducted during 15-min sessions, once a day, 5 days per week. During the first week of training, in order to avoid neophobia to the sweet-tasting pellets, mice were enclosed in the food arm, and a measured quantity of pellets was the only stimulus available during testing. Number of pellets consumed each of the 5 day habituation period was recorded (Exp. 2.2).

The following 2 weeks, mice were exposed to the T-maze with free access to the three stimuli, each one in a different arm. The location of the reinforcers was counterbalanced between animals in order to avoid arm preference. Data presented correspond to the last day of the first week (Exp. 2.3.). Sessions were videotaped, and a trained observer unaware of the experimental condition manually registered all the parameters. Time interacting with the stimuli was the main dependent measure because it allowed for the evaluation of interactions with the three different stimuli with the same units (i.e., seconds). Time allocation is a useful measure of preference, relative reinforcement value, and response choice (Baum and Rachlin 1969). Additionally, we also recorded total arm entries as a measure of ambulation and general exploration, and number of pellets consumed. After the evaluation of spontaneous preference, mice were trained during 2 more weeks, and for each week there were 4 days of baseline plus a testing session in which animals received a TBZ injection (VEH or 8 mg/kg TBZ) in a randomized order. Time interacting with each stimulus, total number of entries, and pellets consumed were also recorded (Exp. 3.1. and 3.2.). All these measures and doses were based on previous studies (Correa et al. 2016, 2020; López-Cruz et al. 2018; Carratalá-Ros et al. 2020, 2021a, 2021b).

### Immunohistochemistry for CDNF

Different groups of animals were perfused at different ages (6, 16, or 29 weeks old) to analyze the immunoreactivity of CDNF in Nacb Core (Exp. 4). Animals were anesthetized with CO_2_ and transcardially perfused using 0.9% physiological saline for 5 min, followed by perfusion with 3.7% formaldehyde for 5 min, and later the brains were extracted. The brains remained in 3.7% formaldehyde overnight and were moved to 30% sucrose cryo-protectant. Forty-micrometer brain slices were cut using a cryostat and non-specific binding sites were blocked with a solution of 3.0% H_2_0_2_ for 30 min at room temperature, followed then with 3.0% bovine serum albumin and 0.1% Triton X-100 in PBS for 1 h at room temperature. CDNF immunoreactivity was stained with a polyclonal rabbit antibody for CDNF (1:200; Invitrogen). This antibody was dissolved in a solution that contained 3.0% bovine serum albumin and 0.1% Triton X-100 in PBS for 24 hr incubation on a rotating shaker at 4°C. After the primary antibody, the slices were rinsed in PBS (3 times for 5 min) and incubated in the secondary antibody, HRP-conjugated anti-rabbit (DAKO) for 90 min on a rotating shaker at room temperature. Finally, slices were washed for 3 times during 5 min and revealed with 3′ diaminobenxidine chromogen. The slices were then mounted to gelatin-coated slides, air-dried, and coverslipped using Eukitt quick-hardening (Sigma-Aldrich). The tissue was then examined by light microscopy equipped with a Leica DFC 450C camera (Leica Microsystems). Quantification of the number of cells that express CDNF immunoreactivity was performed taking photos of sections with a 20× (0.125mm^2/field) objective (Eclipse E600; Nikon) using the light microscope and captured digitally using LASX software. ImageJ software (NIH, Bethesda, USA, http://imagej.nih.gov/ij) was used to quantify cells positively labeled for CDNF, and a macro written to automate particle counting within the region of interest. The size of the region of interest was 218,026 μm^2^. Cell counts were at levels that correspond to 0.98–1.34 mm anterior to bregma (Paxinos and Franklin 2004). For each animal, at least three sections were selected and counts were averaged across slides and sections.

### Statistical analyses

Normally distributed and homogenous data (according to Kolmogorov-Smirnov test) were analyzed by analysis of variance (ANOVA) or Student’s *t*-test. For the operant experiments, two-way factorial ANOVA’s (sex × schedule (within factor)) were used to analyze number of lever presses and amount of 10.0% sucrose solution consumed. A two-way factorial ANOVA for the two between factors (sex × sucrose concentration) was used to compare ml consumed during concurrently-free sucrose exposure. In the PROG/Choice study lever presses, 10.0% and 3.0% sucrose consumption were analyzed by way of Student’s *t*-test for independent samples based on sex. Finally, body weight was analyzed using two-way factorial ANOVA (sex × week). Data from RW turns and pellets consumed were analyzed using factorial ANOVA (sex × day) for adolescents and also for middle-aged mice. Further analyses on the number of turns in the RW and the number of pellets consumed in the T-maze on the latest day were analyzed with a two-way factorial ANOVA (age × sex). For the 3-choice-T-maze experiment, the time interacting with each stimulus, the number of pellets consumed, and the total number of entries in compartments were analyzed also using a series of two-way factorial ANOVA (age × sex). In order to study the effect of TBZ in the two ages independently, a series of two-way factorial ANOVA (sex × treatment; within factor) were used for the time spent with each reinforcer, the pellets consumed, and total number of entries. Sidak’s and Tukey’s tests were used as a post hoc analysis when the interaction was significant. Finally, a two-way factorial ANOVA (sex × age) was used initially to study changes in CDNF immunoreactivity, and in addition one-way ANOVA were used to explore age differences on each sex. All data were expressed as mean ± SEM, and significance was set at *p* < 0.05. Statistical analyses were performed with SPSS Statistics 28, and GraphPad PRISM 8 was used to make the graphs.

## Results

### Experiment 1. Assessment of sex differences in operant tasks that require high effort for fluid sucrose

A total of 51 mice (25 males and 26 females) were used during all the studies of experiment 1.

#### Experiment 1.1. Impact of different ratio requirements on operant performance for 10.0% sucrose

**Fig. 2:**
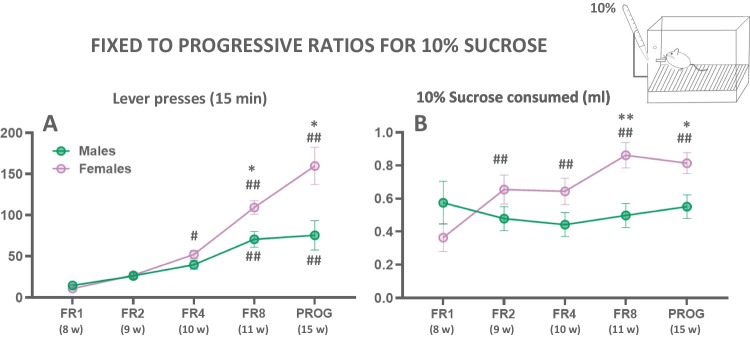
Impact of sex on operant performance across different ratio schedules performed in consecutive weeks. Lever presses (**A**) and 10.0% sucrose intake (**B**). Sessions lasted 15 min. Bars represent the mean ± SEM number of lever presses or ml sucrose consumed. *p<0.05, **p<0.01, significant differences between sexes. ^#^*p*<0.05, ^##^*p*<0.01 significantly different from FR1 in each sex

In order to study sex differences in operant performance on the progression through the different schedules (FR1, FR2, FR4, FR8, and PROG), two-way factorial ANOVAs (sex × schedule) were used (Fig. 2(A, B)). With the analysis of number of lever presses, there was a main effect of sex (*F*(1,49) = 7.60; *p* < 0.01), a main effect of the schedule (*F*(4,196) = 50.30; *p* < 0.01), and a significant sex x schedule interaction (*F*(4,196) = 8.55; *p* < 0.01). Sidak’s post hoc test showed that on the FR8 and PROG schedules, females pressed significantly more than males (*p* < 0.05 for both). Moreover, Sidak’s test revealed that in males, the numbers of FR8 and PROG lever presses where higher than those on the FR1 schedule (*p* < 0.01). For females, the post hoc tests showed differences were seen comparing FR1 and FR4 schedules (*p* < 0.05), and responding increased further when the mice were shifted to FR8 and PROG schedules (*p* < 0.01; see Fig. 2(A)). Thus, these data indicated that mice work harder to obtain the reinforcer as the schedule required more effort. Females worked harder than males to get access to the reinforcer, especially as the task became more effort demanding.

The same statistical analyses were used to study the amount of 10.0% sucrose solution consumed after completing the programmed ratios across the operant sessions. The two-way factorial ANOVA showed no main effect of sex (*F*(1, 49) = 2.56; *p* = 0.12), but a main effect of the operant schedule (*F*(4,196) = 7.04; *p* < 0.01), and a significant interaction (*F*(4,196) = 9.95; *p* < 0.01). Multiple comparisons using Sidak’s test revealed significant differences (*p* < 0.01) in the amount of sucrose solution females consumed for all schedules compared with FR1. Thus, females were more efficient at consuming sucrose than males when their operant performance was the same. Moreover, there was a difference between males and females in the amount of 10.0% sucrose solution consumed in the more effortful schedules (FR8, *p* < 0.01 and PROG *p* < 0.05; data shown in Fig. 2(B)). Females pressed the lever more times and also consumed higher amounts of 10.0% sucrose solutions than males when the task became more effort demanding. Interestingly, males kept a constant amount of sucrose consumption across schedules.

#### Experiment 1.2. Assessment of sex differences in the concurrent free sucrose preference test: 3.0 vs 10.0% concentrations

**Fig. 3:**
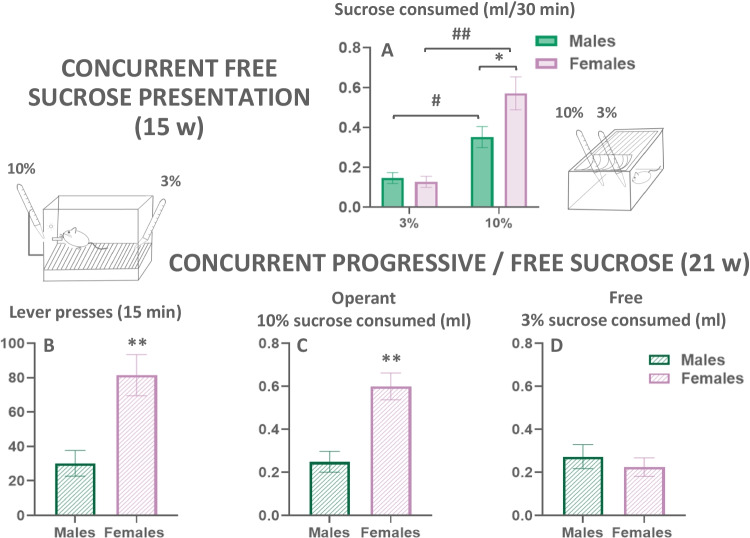
Upper: Effect of concurrent free access presentation of two concentrations of sucrose (10.0 and 3.0%) on volume consumed in a 30-min session in both sexes (**A**). Bars represent mean ± SEM of ml sucrose consumed. *p<0.05 significant differences between sexes. ^#^*p*<0.05, ^##^*p*<0.01 significant differences between concentrations. Lower: Effect of introducing a free option (3.0% sucrose) on PROG lever presses (**B**), operant-dependent 10.0% sucrose intake (**C**), and free 3.0% sucrose intake (**D**). Bars represent the mean ± SEM number of lever presses or ml consumed in an operant session of 15 min. **p<0.01, significant differences between sexes

In order to make sure that these (3.0% and 10.0%) two concentrations of sucrose later used in the PROG/Choice procedure were discriminably different for mice, we evaluate the amount consumed by both sexes under no-effort restrictions in a home cage (Fig. 3(A)). Two-way ANOVA showed a main effect of sucrose concentration (*F*(1,49) = 37.23; *p*< 0.01), no significant main effect of sex (*F*(1, 49) = 3.49; *p* = 0.06), and a significant interaction (*F*(1,49) = 4.99; *p*< 0.05). Sidak’s test revealed that females consume higher amounts of 10.0% sucrose solution compared to 3.0% (*p* < 0.01), and the same occurred in males (*p*< 0.05). Moreover, females consumed more 10.0% sucrose than males (*p*< 0.05). These data indicate that both males and females did not differ in preference for higher concentrations of sucrose under concurrent and free access presentation, but females consumed higher amounts of the most preferred solution.

#### Experiment 1.3. Impact of concurrently freely available 3.0% sucrose solution on progressive ratio performance for 10.0% sucrose solution

After performing on the PROG schedule, mice were trained on a PROG/Choice condition in which they can press the lever in order to obtain the higher-preferred reinforcer (10.0% sucrose solution) and they can also drink a less-preferred reinforcer (3.0% sucrose solution) that is concurrently and freely available during the operant session (Fig. 3(B–D)). A series of *t*-tests for independent samples were used to compare the number of lever presses and the amounts of the 10.0%, and 3.0% sucrose solutions consumed comparing male and female mice. As for lever presses, females pressed the lever more than males (*t* = 3.57; *p* < 0.01; Fig. 3(B)), and consumed higher amounts of 10.0% sucrose solution (*t* = 4.39; *p* < 0.01; Fig. 3(C)), but there was no difference in the amount of 3.0% sucrose solution consumed by males and females (*t* = −0.69; *p* = 0.49; Fig. 3(D)). Thus, females worked harder than males to obtain the high concentration of sucrose even when they had another option that required less effort.

#### Experiment 1.4. Bodyweight changes across the stages of operant testing

**Fig. 4:**
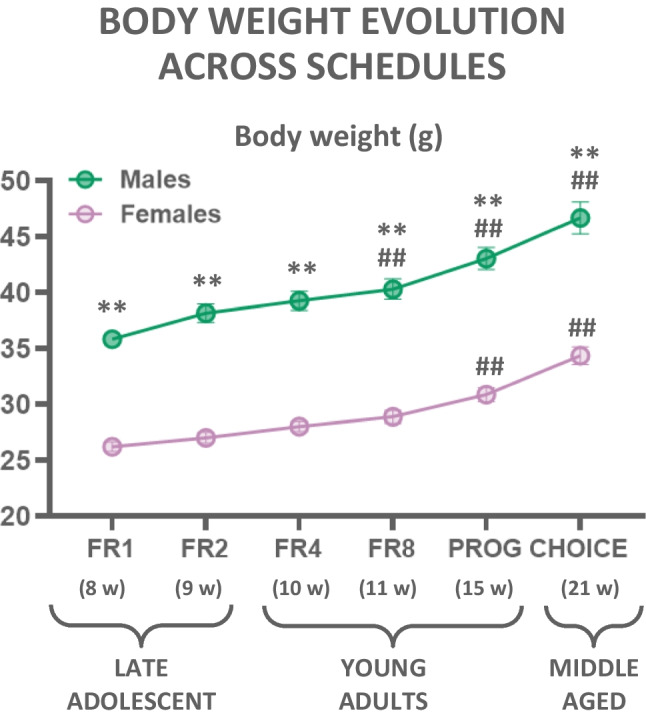
Body weight of both sexes across time. Data represent mean + SEM of body weight in grams during the week that corresponded with the operant data represented. **p<0.01 significant differences between sexes. ^##^*p*<0.01 significant differences with the FR1 week

Finally, bodyweight measures were analyzed to compare sex differences across weeks using a two-way factorial ANOVA. Data in each point represent average body weight during the week in which the operant data were obtained for all the schedules used (FR1 to PROG/Choice; see Fig. 4). Results revealed a main effect of sex (*F*(1,49) = 119.46; *p* < 0.01), a main effect of week (*F*(5,245) = 220.33; *p* < 0.01), and a significant interaction (*F*(5,245) = 4.45; *p* < 0.01). Post hoc analysis using Sidak’s test revealed differences between males and females on body weight in all schedules (*p* < 0.01). Moreover, males increased body weight significantly starting when they were in the week in which the schedule was FR8 compared to week FR1 (*p* < 0.01 for all of them). However, females increased body weight when they reached PROG compared to FR1 (*p* < 0.01 for all the following schedules). Thus, these data indicate that males weighed more than females, and weight increased in both sexes over time, although males increased earlier during training. The percentage of weight gain at the end of the experiments did not differ between sexes (males gained around 22.38%, while females gained 23.22%).

### Experiment 2. Assessment of sex differences on preferences for reinforcers that require effort: studies at different ages

#### Exp. 2.1. Voluntary running in a RW under no-choice conditions

**Fig. 5:**
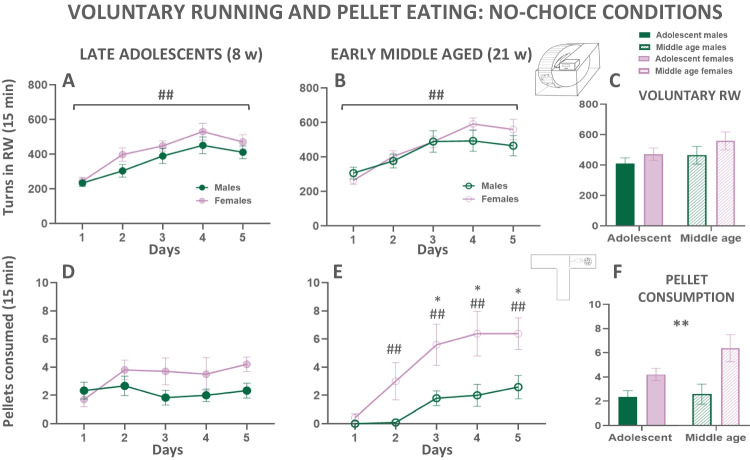
Left: Spontaneous running wheel activity or sweet food intake in naïve adolescent (**A, D**) and naïve middle-aged (**B, E**) male and female mice on 5 consecutive days. Right: Comparison of both ages and both sexes when animals have already reached a stable baseline of voluntary RW (**C**), and sweet food consumed (**F**) on day 5, when animals were already habituated. Data are expressed as mean ± S.E.M of number of turns or pellets consumed during a session of 15 minutes. ^##^*p*<0.01 statistically significant main effect of session, or significant differences from day 1 in the same sex. *p<0.05 significant difference between sexes, **p<0.01 statistically significant main effect of sex

In order to study the preference for voluntary running at different ages and on both sexes, different groups out of the total of 51 animals were used: 12 adolescent males, 15 middle-aged males, 12 adolescent females, and 12 middle-aged females. Two independent two-way factorial ANOVAs (sex × session) were used to compare the number of turns in the RW in adolescent and in middle-aged mice (Fig. 5(A–C)). For the adolescent group, the analyses revealed no effect of sex (*F*(1, 22) = 2.03; *p* = 0.17), and no significant interaction (*F*(4,88) = 0.77; *p* = 0.54). However, there was a significant main effect across training session days on the number of turns (*F*(4,88) = 29.46; *p* < 0.01; Fig. 5(A)).

In the middle-aged mice, the pattern of effects was very similar. The two-way factorial ANOVA revealed no effect of sex (*F*(1,25) = 0.38; *p* = 0.55), and no significant interaction (*F*(4,100) = 2.18; *p* = 0.08), but a main effect of training session days on the number of turns (*F*(4,100) = 26.13; *p* < 0.01; Fig. 5(B)). These results indicate that both sexes are equally active in the RW, and both groups increased running across sessions.

Once baseline levels of voluntary running were stable, we compared both sex and age on day 5. A two-way factorial ANOVA (age × sex) was used and it revealed no effect of sex (*F*(1,47) = 2.22; *p* = 0.14), no effect of age (*F*(1,47) = 1.91; *p* = 0.17), and no significant interaction (*F*(1,47) = 0.11; *p* = 0.74; Fig. 5(C)). These data indicate that mice are equally active in engaging in this reinforcing activity independently of sex and age.

#### Exp. 2.2. Voluntary sweet pellet intake under no-choice conditions

One of the variables evaluated on the T-maze was the number of pellets consumed during the first week in which mice were enclosed in the food compartment to get habituated to the taste of the new food. Four groups of mice were evaluated: 12 adolescent males, 12 middle-aged males, 12 adolescent females, and 12 middle-aged females. Two-way factorial ANOVA (sex × session) was used to compare the number of pellets that males and females consumed during the 5 days of *ad libitum* exposure to the high carbohydrate pellets for the adolescent and a separate ANOVA for the middle-aged group (see Fig. 5(D–F)). In the adolescent group, there was no significant effect of sex (*F*(1,20) = 2.58; *p* = 0.12), no significant effect of days (*F*(2,57) = 1.96; *p* = 0.13), and no significant interaction (*F*(4,80) = 2.07; *p* = 0.09; Fig. 5(D)).

In the middle-aged group, there was a significant effect of the sex factor (*F*(1,22)= 7.44; *p* < 0.01), a main effect of days (*F*(2,61) = 18.26; *p* < 0.01), and also a significant interaction (*F*(4,88) = 3.24; *p* < 0.05; Fig. 5(E)). Tukey’s post hoc test showed that females increased consumption of pellets starting on the second day and for all the sessions afterwards (*p*<0.01) compared to the first day, and they also consumed significantly more pellets than males on the third, fourth and fifth days (*p*<0.05). However, middle-aged males did not significantly increase intake on any given day when compared with the first day. Thus, these data suggest that in the adolescent period, there were no differences in the preference for consuming a high carbohydrate food pellets between males and females, but with age, females consumed higher amounts of that kind of food than males.

In order to study differences in the preference for high carbohydrate food considering both, sex and age conditions, a two-way ANOVA (age x sex) for the number of pellets consumed during the last day of T-maze habituation to food was used. The analyses revealed a significant main effect of sex (*F*(1,42) = 12.13; *p* < 0.01), but no significant effect of age (*F*(1,42) = 2.23; *p* = 0.14), and no significant interaction (*F*(1,42) = 1.40; *p* = 0.24; Fig. 5(F)). Therefore, these data reinforce again the observation that females had a stronger tendency to consume the high palatable food compared to males.

#### Exp. 2.3. Differences of sex and age on baseline preferences over the three concurrently presented stimuli (RW, sweet food and neutral odor) in the 3-choice-T-maze task

**Fig. 6:**
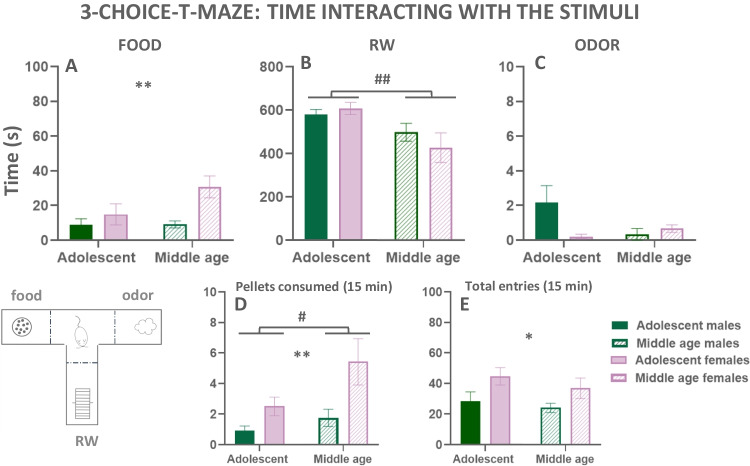
Preference for stimuli with different levels of vigor requirements in the 3-choice T-maze task. Time eating palatable food (**A**), time running (**B**), time sniffing the non-social odor (**C**), pellets consumed (**D**), and motor exploration of the 3-compartments T-maze (**E**). Data are expressed as mean ± S.E.M of time (seconds), number of pellets consumed or number of entries in the 3 compartments during a 15-min session. *p<0.05, **p<0.01 significant main effect of sex. ^#^*p*<0.05, ^##^*p*<0.01 statistically significant main effect of age

After the habituation to the food compartment, the same four groups of mice used in the Exp. 2.2. were trained in the 3-choice-T-maze task (data showed in Fig. 6(A–E)). Two-way ANOVAs (age × sex) were used to compare the time mice interacted with each stimulus. For time eating (Fig. 6(A)), the two-way ANOVA showed a main effect of sex (*F*(1,42) = 7.65; *p* < 0.01), no effect of age (*F*(1,42) = 2.57; *p* = 0.12), and no significant interaction (*F*(1,42) = 2.45; *p* = 0.12). For time running in the RW (Fig. 6(B)), the two-way ANOVA revealed a main effect of age (*F*(1,42) = 8.42; *p* < 0.01), but no effect of sex (*F*(1,42) = 0.24; *p* = 0.62), and no significant interaction (*F*(1,42) = 1.15; *p* = 0.29) either. For time sniffing the odor (Fig. 6(C)), the two-way ANOVA showed no effect of age (*F*(1,42) = 1.51; *p* = 0.23), and no effect of sex (*F*(1,42) = 2.15; *p* = 0.15); nevertheless, a significant interaction was found (*F*(1,42) = 4.27; *p* <0.05). However, Sidak’s post hoc test showed no significant differences between groups.

During the concurrent exposure to the three stimuli, we also recorded the number of pellets consumed in the 15-min session. Analysis using a two-way ANOVA showed a significant main effect of sex (*F*(1,42) = 8.47; *p* < 0.01), and also a significant main effect of age (*F*(1,42) = 4.32; *p* < 0.05), but no significant interaction (*F*(1,42) = 1.33; *p* = 0.25; Fig. 6(D)). Thus, females once again consumed more sucrose pellets than males, and middle-aged adults consumed more than adolescents, even when they had other reinforcers available that were highly preferred like the RW.

Another variable that provided information about behavioral activation in the 3-compartment-T-maze was the total number of entries in compartments as an index of horizontal exploratory locomotion. The two-way factorial ANOVA revealed a main effect of sex (*F*(1,42) = 6.77; *p* < 0.05), but no effect of age (*F*(1,42) = 1.12; *p* = 0.29), and no significant interaction (*F*(1,42) = 0.10; *p* = 0.75; Fig. 6(E)). These data suggest that despite eating more, females were also more active explorers than males.

#### Experiment 3. Effect of TBZ on preference for active reinforcers assessed in the 3-choice-T-maze task in male and female mice of different ages

##### Exp. 3.1. Effect of TBZ in adolescent mice

**Fig. 7:**
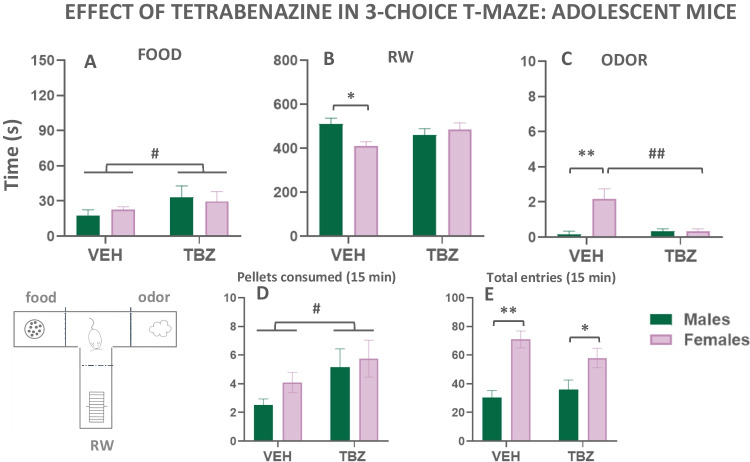
Effect of TBZ (vehicle or 8.0 mg/kg) on time eating (**A**), time running (**B**), time sniffing (**C**), pellets consumed (**D**), and entries into compartments (**E**) in adolescent mice of both sexes as measured in the T-maze task. Bars represent the mean ± S.E.M. of accumulated seconds, number of pellets, or number of entries in 15 min. *p<0.05, **p<0.01 significant differences between sexes. ^#^*p*<0.05, ^##^*p*<0.01 significant main effect of treatment or significant differences between VEH and TBZ in the same sex

In order to study the preference for active reinforcers in adolescent mice of both sexes under the effect of the DA depleting agent TBZ, a series of two-way factorial (treatment × sex) ANOVAs were used (data represented in Fig. 7(A–E)). The adolescent animals exposed to TBZ were the same animals used in the 3-choice-T-maze task during the Experiments 2.2 and 2.3. (12 adolescent males and 12 adolescent females). For the independent variable time spent eating, the two-way ANOVA showed a significant main effect of treatment (*F*(1,22) = 4.186; *p* < 0.05), but no significant effect of sex (*F*(1,22) = 0.01; *p* = 0.94), and no significant interaction (*F*(1,22) = 0.62; *p* = 0.44; Fig. 7(A)). For the time animals spend running in the RW, the statistical analyses revealed no significant main effect of treatment (*F*(1,22) = 0.254; *p* = 0.62) and no significant effect of sex (*F*(1,22) = 1.67; *p* = 0.21), although there was a significant interaction (*F*(1,22) = 7.285; *p* < 0.05). Sidak’s test revealed that under vehicle conditions, females spent less time running than males (*p* <0.05). Also, there was a tendency for males to show reduced running on TBZ compared to vehicle, but females tended to show the opposite pattern (Fig. 7(B)). The two-way ANOVA for the time adolescent animals spent sniffing revealed a significant main effect of treatment (*F*(1,22) = 6.18; *p* < 0.05), a significant main effect of sex (*F*(1,22) = 11.48; *p* < 0.01), and also a significant interaction (*F*(1,22) = 8.89; *p* < 0.01). Sidak’s test showed that under vehicle conditions, females spent more time sniffing the odor than males (*p* < 0.01), and the administration of TBZ decreased the time females spent sniffing the odor (*p* < 0.01; Fig. 7(C)). These data suggest that under vehicle conditions, females spent less time running but more time sniffing the odor than males. More importantly, the preference for active reinforcers in both sexes was not affected in adolescence by the administration of TBZ, although it did increase the time that animals spent eating the high carbohydrate food.

This last point was confirmed when we evaluated the effect of TBZ on the amount of high carbohydrate food consumed. The two-way ANOVA revealed a significant main effect of treatment (*F*(1,22) = 5.62; *p* < 0.05), but no significant effect of sex (*F*(1,22) = 1.03; *p* = 0.32), and no significant interaction (*F*(1,22) = 0.29; *p* = 0.59; Fig. 7(D)).

Finally, as a measure of activation, the two-way ANOVA for the total number of entries showed a significant main effect of sex (*F*(1,20) = 18.11; *p* < 0.01), no significant effect of treatment (*F*(1,20) = 0.66; *p* = 0.42), but a significant interaction (*F*(1,20) = 4.10; *p* < 0.05). Sidak’s test showed that females did more entries than males under both vehicle (*p* < 0.01) and also TBZ conditions (*p* < 0.05; Fig. 7(E)).

##### Exp. 3.2. Effect of TBZ in middle-aged mice

**Fig. 8:**
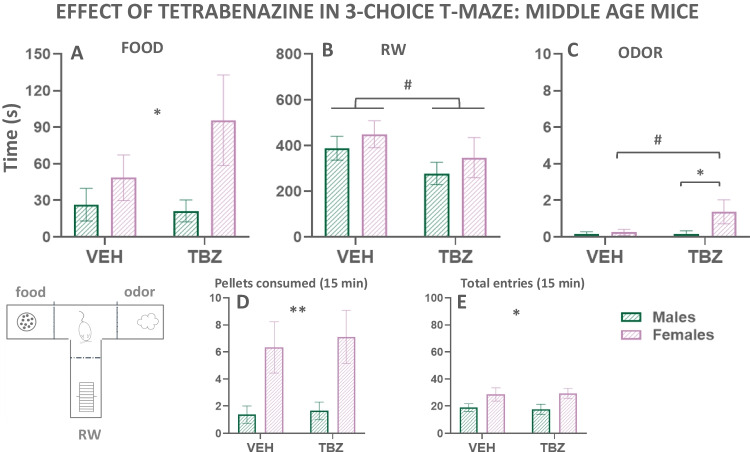
Effect of TBZ (vehicle or 8.0 mg/kg) on time eating (**A**), time running (**B**), time sniffing (**C**), pellets consumed (**D**), and entries into compartments (**E**) in middle-aged adult mice of both sexes as measured in the T-maze task. Bars represent the mean ± S.E.M. of accumulated seconds, number of pellets, or number of entries in 15 min. *p<0.05, **p<0.01 statistically significant main effect of sex or differences between sexes in the same treatment. ^#^*p*<0.05 significant main effect of treatment or significant differences between VEH and TBZ in the same sex

A series of two-way factorial ANOVAs (treatment × sex) were used to evaluate the effect of TBZ administration in the middle-aged mice. The middle-aged mice exposed to TBZ injections are the same animals used in experiments 2.2. and 2.3. (12 middle-aged males and 12 middle-aged females; Fig. 8(A–E)). For time eating the high carbohydrate food, the statistical analyses revealed a significant main effect of sex (*F*(1,18) = 4.77; *p* < 0.05), but no significant effect of treatment (*F*(1,18) = 1.62; *p* = 0.22), and no significant interaction (*F*(1,18) = 2.49; *p* = 0.13; Fig. 8(A)). The two-way ANOVA for the time running showed a significant main effect of treatment (*F*(1,18) = 5.35; *p* < 0.05), but no significant effect of sex (*F*(1,18) = 0.79; *p* = 0.38), and no significant interaction (*F*(1,18) = 0.006; *p* = 0.94; Fig. 8(B)). For time sniffing, the statistical analyses revealed no significant main effect of sex (*F*(1,18) = 3.56; *p* = 0.07), although there were a significant main effect of treatment (*F*(1,18) = 5.24.; *p* < 0.05), and a significant interaction (*F*(1,18) = 5.24; *p* < 0.05). Sidak’s test showed that TBZ administration increased the time that females spend sniffing the odor (*p* < 0.05) compared to vehicle, and also under the effect of TBZ, females sniff the odor more than males (*p* < 0.05; Fig. 8(C)). These results suggest that TBZ administration reduced the preference for the active behavior of wheel running in middle-aged mice. Moreover, female mice seem to compensate by increasing time eating and time sniffing the odor.

The total number of pellets consumed were also evaluated using a two-way ANOVA and it revealed a significant main effect of sex (*F*(1,18) = 8.35; *p* < 0.01), but no significant effect of treatment (*F*(1,18) = 1.07; *p* = 0.31), and no significant interaction (*F*(1,18) = 0.25; *p* = 0.62; Fig. 8(D)). Thus, middle-aged females not only spent more time eating, but also consumed higher amounts of high carbohydrate food than middle-aged males.

Finally, the statistical analyses for the total number of entries showed a significant main effect of sex (*F*(1,22) = 4.52; *p* < 0.05), but no significant effect of treatment (*F*(1,22) = 0.01; *p* = 0.91), and no significant interaction (*F*(1,22) = 0.31; *p* = 0.59; Fig. 8(E)). Thus, females were more active exploring all the compartments than males in the middle-aged period.

### Experiment 4. Impact of age and sex on CDNF immunoreactivity in Nacb Core

**Fig. 9:**
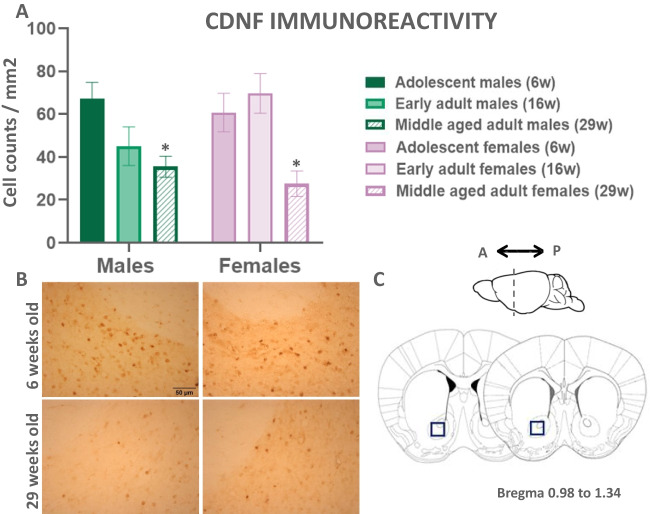
(**A**) CDNF immunoreactivity in Nacb Core at different ages in both sexes. Bars represent the mean ± S.E.M. of positive cell counts per mm^2^. *p<0.05 significantly different from 6w old animals. (**B**) Representative pictures (40×) showing expression of CDNF immunoreactivity in the Nacb Core of males and females at 6 and 29 weeks. Scale bar represents 50 μm. (**C**) Diagram of coronal sections with bregma coordinates showing location of Nacb Core taken from Paxinos and Franklin (2019)

In order to study potential differences due to age (6, 16, and 29 weeks old) and sex on CDNF immunoreactivity in the Nacb Core, a new group of naïve animals was used (*N* = 36; 6 animals/age/sex; see Fig. 9(A–C)). A two-way ANOVA was used and it revealed a significant main effect of age (*F*(2, 30) = 9.76; *p* < 0.01), but no significant effect of sex (*F*(1, 30) = 0.29; *p* = 0.59), and no significant interaction (*F*(2, 30) = 2.81; *p* = 0.08). As no differences were found in the sex factor, but the interaction approached significance, two different one-way ANOVA were performed to study the effect of age on each sex. In males, the statistical analysis revealed a significant main effect of age (*F*(2, 15) = 4.96; *p* < 0.05). Sidak’s test revealed that the immunoreactivity of CDNF in males only decreased at 29 weeks of age compared to the 6 weeks of age (*p* < 0.05). In females, the one-way ANOVA also showed significant differences (*F*(2, 15) = 7.35; *p* < 0.01), and Sidak’s test also revealed that females presented lower CDNF immunoreactivity at 29 weeks of age compared to 6 weeks of age (*p* < 0.05; Fig. 9(A)).

## Discussion

These experiments were designed to explore differences based on sex and age in mice performing on tasks involving effort-based decision-making. We used several tasks to evaluate the innate preferences for high carbohydrate food or sucrose solutions, and we examined the willingness to exert effort in order to obtain the sweet reinforcers. Moreover, we evaluated differences in relative preferences for activities that require vigor, like RW, when presented in competition with other reinforcers that are accessed by more sedentary behaviors, such as intake of sweet food. In addition, we explored the impact of age on parameters related to Nacb DA function (i.e., the neurotrophic factor CDNF), and assessed the impact of TBZ on vigor preferences at different ages.

The first result to highlight in the present study is that females are different from males in their level of responding on those operant tasks that require more work output (FR8 and PROG). Moreover, while the male mice maintained a level of 10% sucrose consumption as a result of their work output across all the schedules, females achieved a higher amount of fluid consumed across the FR2, FR4, FR8, and PROG schedules, indicating that they were more efficient at drinking when the bottle was available after completion of the ratio (Fig. 2(B)). Thus, males seem to minimize their effort to a level that maintains their sucrose intake, while females maximize their sucrose intake. Different studies support the evidence that female mice consume more sucrose solutions than males (Ren et al. 2020; Wei et al. 2021a, 2021b), both under free conditions or after operant settings. In FR1 schedules of operant responding to obtain a sugar solution, males and females had similar performance, but when the task became more effort-demanding (FR4 or PROG), females emitted more lever presses, indicating higher persistence in sugar-seeking behavior (Wei et al. 2021a).

Because the main purpose of the present work was to observe potential differences in effort-based decision-making, operant responding had to be evaluated under choice conditions, but before the PROG/Choice sessions, mice had 2 days of concurrent presentation of two sucrose solutions (3.0% and 10%) freely accessible in a home cage (Fig. 3(A)). Both sexes clearly preferred the most concentrated solution, but females consumed significantly more of the high concentration than males. The PROG/choice studies (Figs. 2(A) and 3(B)) showed that the presence of the free option reduced the amount of work output by around 50% in both sexes. Although the difference in lever pressing between males and females is still sustained, both sexes did not differ in terms of the intake of the freely available low-sucrose solution (Fig. 3(D)). Interestingly, in the PROG/Choice task, both males and females ended up consuming a total volume of fluid (0.3 plus 10%; Fig. 3(C, D)) that was about the same as the volume consumed when there was no free option (see Fig. 2(B)); males consumed a total volume of 0.5 ml and females 0.8 ml. Despite the higher consumption of the more concentrated sucrose solution, female and male mice did not differ in terms of body weight gain across the experiment (22.38% of increment in males and 23.22% in females comparing FR1 with PROG/choice).

Thus, performance on schedules with lower work requirements (FR1-FR4) was virtually identical in both sexes, and differences in performance emerged at the most effort-demanding tasks (FR8 and PROG). The schedules with low work requirements were performed mainly when mice were in their late adolescence. However, the sexes showed differences when they were young adults (11–16 weeks old), and especially when they were transitioning between young adults to the early stages of middle age (21 weeks old). This information is relevant because age could be a mediating variable when using operant procedures that require long periods of training. Thus, differences between sexes observed in these difficult operant procedures could be due to aging, rather than to work demands. Other studies using C57 mice of a comparable age to our young animals that were tested with effort-choice procedures (Dieterich et al. 2021) found a similar pattern of results in a Y-maze barrier task in which the high-density (HD) arm had double the number of those same pellets and animals had to climb a barrier to get access to them or go to an unobstructed arm with half the pellets. These authors found that females (12–13 weeks old) selected the HD arm more often than males (Dieterich et al. 2021). Thus, young females worked harder than males to get access to food reinforcers. This appears to support the idea that sex differences in effort are age dependent. However, in a PROG task reinforced with food pellets, male and female mice 13 weeks old did not show differences in lever pressing (Dieterich et al. 2021), contrary to our PROG results in which sucrose solutions were used as the reinforcer in young and middle-aged mice.

To be able to account for age and to introduce a totally different experimental setting than the operant tasks, in the second part of these studies, we used a paradigm that assesses decision-making processes based on effort and vigor, but that is based on spontaneous preferences of mice, and it requires very little training (Correa et al. 2016, 2020; López-Cruz et al. 2018; Carratalá-Ros et al. 2020, 2021a, 2021b). Thus the 3-choice-T-maze task could be more suitable for comparing mice of different ages. In addition, although it uses palatable food (50% carbohydrate pellets), this option competes with non-palatable options; a RW that is highly preferred, and a very neutral option, which is an olfactory stimulus (a fruit essence). The two ages chosen were based on the starting and ending age of the animals in the previous operant experiment (8-week-old and 21-week-old animals), and which correspond to late adolescence and early middle age (Burke and Miczek 2013; McWain et al. 2022).

Before starting the choice procedure, we evaluated how males and females that were naïve to the RW, and also to the type of sweet food that they will have in the 3-choice-T-maze task, interacted with these two stimuli. When mice were assessed on a voluntary RW with no other option, all groups of animals increased performance across 5 sessions (Fig. 5(A–C)), and there were no differences between sexes or ages. These data agree with previous results showing that CD1 mice do not show sex differences in spontaneous RW activity when living in environmentally enriched housing conditions across weeks or at different times during the circadian cycle (Aujnarain et al. 2018). However, other studies have observed that mice of both sexes are at their peak of voluntary RW activity at 2 months of age (the same age that our late adolescent mice) compared to older ages, and although females achieved higher running velocities than males at this younger age, they do not have shorter running times (Bartling et al. 2017). In that study, mice were evaluated until senescence and results showed reduced RW activity at a much older age than the one studied in the present work (Bartling et al. 2017). In terms of high carbohydrate pellet consumption under no-choice conditions in the habituation period of the T-maze, we observed again how middle-aged females consumed more than middle-aged males, and females of any age ate more than males (Fig. 5(D–F)). Thus, we consistently observed a higher intake of sweet foods or fluids as females get older compared to males.

In the 3-choice-T-maze task (Fig. 6(A–E)), the present data are the first to show in female mice, as was previously shown in males (Correa et al. 2016, 2020; López-Cruz et al. 2018; Carratalá-Ros et al. 2020, 2021a, 2021b), a strong preference for the RW compared to more sedentary reinforcers. There were no differences based on sex in the selection of this vigor-requiring reinforcer. Moreover, in the present studies, there were significant differences between ages; time running in the RW was reduced in middle-aged animals independently of sex, and they compensated by eating more than younger animals, thus showing a more sedentary profile. Moreover, in terms of sweet reinforcers, a sex effect emerged again. Females have a higher relative preference for this palatable food compared to males. Independently of age, females spent more time eating and consumed more pellets, with a clear tendency of the older females to have more intake (Fig. 6(A, D)), corroborating the results in the operant and free access conditions.

Thus, age emerged as a differentiating factor in terms of relative preferences for vigorous activities under choice situations. The Nacb Core is a central structure in the regulation of effort-based decision-making, and DA is a key neuromodulator for these processes (Salamone and Correa 2002, 2012, 2022). DA neurons are particularly vulnerable to aging (McWain et al. 2022). It has been demonstrated that DA receptor expression (D1 and D2) and DA release and activity in the Nacb reaches its highest point during mid-adolescence and young adulthood respectively, declining during adulthood (Burke and Miczek 2013; Karkhanis et al. 2019; Huang et al. 1995; Pitts et al. 2020; Santiago et al. 1993; Stamford 1989; Tarazi et al. 1998; Tarazi et al. 1999), although the peak in DA receptors is much less dramatic in females than males (Andersen et al. 1997). Aged male and female mice have lower DA and DOPAC in Nacb compared to younger mice (Winner et al. 2017), and pre- and postsynaptic functions in Nacb (e.g., long-term depression) in mice decrease with age (Wang 2008).

Thus, depletion of DA with the VMAT-2 blocker TBZ, at a dose that has been demonstrated previously in male mice to deplete DA in ventral striatum (López-Cruz et al. 2018), was used to study potential differences in effort-based decisions in the 3-choice-T-maze task between males and females of the two ages (8 and 21 weeks old). Previous studies in our laboratory, using only males of an age intermediate to those used in the present work, evaluated the effect of TBZ and demonstrated that this drug induced a low-effort bias. Animals decreased time spent running, the most vigorous activity, and instead increased time spent eating, a much more sedentary one (López-Cruz et al. 2018; Carratalá-Ros et al. 2020, 2021a, 2021b). The present results did not have an identical pattern in either of the two ages. For adolescent mice, TBZ did not affect time running. However, at middle age, mice were more vulnerable to the impairing effects of TBZ on vigor-based decision-making, since TBZ was able to decrease time running compared to vehicle. Brain DA circuits involved in behavioral activation seem to be at a maximal performance level in adolescence and early adults (Burke and Miczek 2013; Karkhanis et al. 2019; Huang et al. 1995; Pitts et al. 2020; Santiago et al. 1993; Stamford 1989; Tarazi et al. 1998; Tarazi et al. 1999). Thus, vigorous responses in late adolescents seem to be less affected by fluctuations in DA levels: either by drugs that reduce DA (in the present results) or by drugs that increase DA levels such as bupropion (Redolat et al. 2005). The dose of TBZ used did not alter horizontal exploration (entering and exiting the three compartments) of the T-maze at any age (Figs. 7(E) and 8(E)). Thus, the effects of TBZ on vigor preference in older animals did not appear to be general impairments in locomotion. By depleting DA, TBZ seems to be producing anergia (i.e., a reduction of behavioral activation in the face of other more sedentary choices), rather than affecting the primary reinforcing effects of sweet food, since time spent eating and amount of food consumed actually increased among the late adolescent and young animals (present and previous results López-Cruz et al. 2018; Carratalá-Ros et al. 2020, 2021a, b). Moreover, TBZ did not suppress food intake in middle-aged animals. Perhaps because the level of sweet food intake was already very high in the older females, anergia in females receiving TBZ was demonstrated as a decrease in RW activity, and a small increase in exploration of the most passive reinforcer (time sniffing a neutral odor).

To account for changes in DA function with age, in the final phase of the study, we assessed CDNF immunoreactivity in Nacb Core of both sexes at a range of ages that included the ones used in the present behavioral experiments. CDNF has been found in DA neurons, and is thought to promote the survival of DA terminals due to processes that increase with age (Conde and Streit 2006; Lindahl et al. 2020). The present results demonstrate for the first time that this factor is reduced with age in mice of both sexes under basal conditions in this nucleus critically involved in regulating effort-based choices. This reduced protection in the aging Nacb Core may be related to the effect of the VMAT-2 blocker on reducing time interacting with the most vigorous reinforcer in the middle-aged mice. In rodent models, it has been observed that with increasing age, several markers of neural protection such as BDNF are reduced in different brain regions (Katoh-Semba et al. 1998). More specifically and consistently with our results, the glial cell line–derived neurotrophic factor (GDNF), known to modulate and functionally enhance the DA system (Airavaara et al. 2004), has been involved in aging impairments. Heterozygous male GDNF+/− mice exhibited an accelerated age-related decline in horizontal activity and TH immunostaining in substantia nigra, compared to WT mice GDNF+/+ (Boger et al. 2006). Similar to our operant results, Griffin et al. 2006 showed that 6-month-old GDNF+/− female mice with significantly less GDNF protein content in striatum than the WT counterparts emitted slightly more FR1 operant responses for sucrose. However, in that study, there were no males.

Thus, in our studies, females consumed more sweet foods than males at all ages tested and across multiple access conditions. This sex-dependent difference in sweet consumption increased as the mice got older. Thus, between 16 and 21 weeks of age, females consumed more of the sucrose solution used for operant reinforcement, and they ate more free available pellets under no-choice and choice options in the T-maze. It has long been proposed that female mice of different strains consume more highly caloric food and drinks (alcohol and sucrose) than males, probably due to metabolic differences (Short et al. 2006; Satta et al. 2018; Zhou et al. 2019). Although they also seem to consume palatable solutions that do not contain calories (saccharine), differences between sexes in this case seem less pronounced (Zhou et al. 2019). It is important to highlight that our female mice do not differ from males in preference for vigorous activities in the T-maze, they do not run for longer periods of time, but they do explore the three compartments more than males, and the main differences emerged when the vigorous instrumental response allowed a higher intake of sweet reinforcers. Thus, it is possible that these sex- and age-related aspects of appetite for caloric drinks can be modulated by DA in other brain areas. It seems that, while DA in striatal areas decreases with age (Winner et al. 2017), DA concentration and DA receptor density increases in the anterior pituitary gland. Old and middle-aged females have higher levels of pituitary DA than younger females and also than young and middle-aged males (Telford et al. 1987; Kochman et al. 1993). DA levels in this key area for hormonal regulation could regulate the observed differences between males and females on consumption of sweets. For example, DA modulates prolactin (PRL) synthesis and secretion (Fernandez-Ruiz et al. 1989; Mena et al. 1989; Kochman et al. 1993), and DA receptor agonists, via PRL, can regulate blood glucose content (Kirsch et al. 2022; Chien et al. 2023).

In summary, we can conclude that sex and age are important variables to consider when working with mice using tests of effort-based decision-making. Moreover, if effort-based tasks use palatable reinforcers, it is more likely that sex differences will be observed. Thus, further experiments should explore the nature of sex differences using other types of motivational stimuli. In addition, a broader exploration of the impairing effects of DA depleting drugs at different ages in both sexes will give a more comprehensive perspective on sex differences and vulnerability to anergia. Behavioral pharmacology relies on animal models that are primarily validated using the male laboratory rodent. Many researchers solely employ young male animals in studies primarily due to concerns about budgets or time, but also about the impact of variations in the female estrous cycle on behavioral responses. However, ample evidence demonstrates that female rodents are not more variable than rodent males due to hormonal changes (Shansky 2019; Prendergast et al. 2014; Becker et al. 2016; Beery and Zucker 2011). In any case, if chronic treatments need to be implemented, the different phases of the estrous cycle need to be averaged. Moreover, the age at which the animals show deficiencies or start to get treatments is important in some studies.

The knowledge of how similar both sexes are in distinct aspects of motivation is essential for the understanding of the neurophysiology that underlies these processes. Moreover, using both sexes and different ages can help to develop future personalized treatments, especially considering that depression can have different characteristics in women compared to men across the life span. This is especially important since effort-related symptoms are thought to reflect a common set of dysfunctions present not only in major depression, but also in Parkinsonism, drug abstinence, multiple sclerosis, and negative symptoms in schizophrenia (Caligiuri and Ellwanger 2000; Salamone and Correa 2012, 2022; Friedman et al. 2007; Tellez et al. 2008; Tylee et al. 1999; Demyttenaere et al. 2005; Simpson et al. 2012). As it seems with depression (Bjornelv et al. 2011; Dekker et al. 2007), it is possible that sex is a relevant factor in the manifestation of motivational dysfunctions in people with these disorders. Future studies or metanalysis should address this question.

## Data Availability

Any data will be made available upon reasonable request to the corresponding author.
